# Turrill’s Omega: A Heuristic Index for Quantifying the Support of a Taxonomic Hypothesis

**DOI:** 10.3390/biology15151265

**Published:** 2026-08-02

**Authors:** Lorenzo Peruzzi, Jacopo Franzoni, Antonio Giacò

**Affiliations:** 1 PLANTSEED Lab, Department of Biology, University of Pisa, Via Derna 1, 56126 Pisa, Italy; jacopo.franzoni@biologia.unipi.it (J.F.); antonio.giaco@biologia.unipi.it (A.G.); 2 Botanic Garden and Museum, University Museum System, University of Pisa, Via Luca Ghini 13, 56126 Pisa, Italy

**Keywords:** biodiversity, species hypothesis, systematics, taxonomy

## Abstract

Biological research relies on the use of taxa, with species expressed in the form of a Latin binomial. Although taxa are commonly regarded as established entities, they actually represent hypotheses on the distribution of natural biological variation and may change their status as new systematic knowledge becomes available. A long-standing problem affecting systematics and taxonomy, with important consequences for other biological disciplines, is that a binomial itself conveys no information about the strength of the taxonomic hypothesis it represents. Here, we introduce the concept of a standardized heuristic index, named Turrill’s Omega Index (TOI), to estimate the degree of support for a taxonomic hypothesis. Turrill’s Omega Index is a single numerical value calculated by integrating four macro-categories of comparative data: Phenotype, Genealogy, Isolation, and Geography-Ecology. Turrill’s Omega Index provides a practical and reliable framework for quantifying the support for a taxonomic hypothesis, without requiring resolution of the long-standing debate surrounding species delimitation and species concepts. Accordingly, it has the potential to represent a valuable and transformative advance in systematics and taxonomy. In addition, TOI can support conservation decision-making by helping prioritize funding allocation among equally threatened taxa according to their degree of support.

## Simple Summary

In biology, taxa are hypotheses, with species typically expressed as Latin binomials. However, these names convey no information about the strength of the evidence supporting the underlying taxonomic hypothesis. Here, we propose a standardized heuristic metric, the Turrill’s Omega Index (TOI), to estimate the degree of support for a taxonomic hypothesis. Turrill’s Omega Index provides a practical and robust framework that enables both taxonomists and, especially, users of taxonomy to critically evaluate the level of support for taxa, rather than treating binomials as fixed and immutable entities. In addition, TOI may assist conservation decision-making by helping prioritize the allocation of limited resources among equally threatened taxa according to their degree of support.

## 1. What Are the Issues with Scientific Names and Species Concepts?

Systematics and taxonomy are two fundamental disciplines in biology. The former deals with delimiting diversity groups using comparative data, while the latter aims at translating and organizing these units (i.e., taxa) into coherent, hierarchical classification systems [1]. Biological research fully relies on the use of taxa, expressed in the form of a Latin binomial for species. These are often regarded, particularly by users of taxonomy, as established units that are expected to have scientific names that should not change over time (see, e.g., [2,3]).

In reality, taxa are hypotheses on the distribution of biological variation in nature [4] and can be “split” or “lumped” when new systematic knowledge is produced. For instance, if new comparative data do not support the distinction of two species, these are synonymized, i.e., they are lumped into a single species. Conversely, if new data support the distinction of two groups of populations that were previously considered as belonging to the same taxonomic unit, these are split in two different species.

However, *taxonomic* species hypotheses can change also based on the *systematic* species concept adopted (e.g., biological, morphological, ecological, phylogenetic, etc.). The choice of the species concept to apply may depend on the taxonomic group, but also on the researcher’s school of thought. This makes it impossible to completely avoid a degree of subjectivity in the delimitation of natural variation.

By simply reading a scientific name, it is impossible to know whether it is supported by systematic data, both in terms of quantity (e.g., number of different lines of evidence) and quality (e.g., completeness of taxon sampling, correctness of methods). Similarly, it is impossible to infer which of the many species concepts a scientific name is based on, either explicitly or implicitly. Accordingly, it is impossible to determine the support and reliability of a species hypothesis solely by reading its name, without ad hoc research.

To date, there is no tool or index allowing an evaluation of this degree of support. Indeed, due to several biological phenomena (such as apomixis, hybridization, aneuploidy, polyploidy, etc.), systematic investigations are often particularly challenging. Moreover, the so-called “biological species concept”, widely applied by the zoological community, often fails in delimiting plant species [5]. This forces botanists to rely on a plethora of other species concepts, which frequently contrast with each other.

For all these reasons, the terms “taxonomic anarchy” and “taxonomic inflation” are unfortunately widely used in the scientific community [6]. A tool allowing an evaluation of the solidity of any taxonomic hypothesis is urgently needed.

The problem of defining what constitutes a species has been a subject of intense debate since Darwin’s [7] time. He dedicated an entire chapter to an attempt, which was ultimately unsuccessful, to provide a definition for the fundamental unit of its theory. Traditionally, biologists apply a morphological species concept to delimit species, i.e., two populations are considered as distinct species if they exhibit sufficient morphological distinctiveness [8]. This approach is easy, fast, and inexpensive. However, its major disadvantage is that morphology does not always reflect the evolutionary history of a group and can be subjectively interpreted in different ways [9].

Almost a century ago, Turrill [10] already encouraged taxonomists to move beyond “alpha-taxonomy”, based only on morphology, towards an ideal “omega-taxonomy”, by employing as many lines of biological evidence as possible. After Turrill, systematics benefited from the implementation of new approaches, such as cytosystematics and molecular systematics.

The former approach peaked during the 1960s and the 1970s, allowing biologists to formulate taxonomic hypotheses using new evidence such as chromosome number, genome size, and karyotype structure [11]. This approach remains fundamental for a better understanding of plant systematics, since biological phenomena such as dysploidy and polyploidy are particularly common in plants compared with other systematic groups [12].

However, the revolution in the field of systematics started during the ‘80s, when new laboratory techniques and procedures allowing the easy manipulation of DNA sequences (e.g., PCR) were implemented. Due to the vast amount of information contained in DNA molecules, systematists started inferring evolutionary relationships among taxa by reconstructing molecular phylogenies, understanding the paleobiogeography of many taxonomic groups, and investigating population genetic dynamics.

In addition, species concepts that were previously impossible to apply using just morphological or cytogenetic data, such as the evolutionary [13], phylogenetic [14], and cohesion [15] species concepts, started to be adopted to formulate taxonomic hypotheses.

However, the success of molecular systematics contributed during the 1990s to deepening a taxonomic crisis, known as “taxonomic impediment” [16,17,18]. Indeed, taxonomy faced a crisis due to a shortage of experts and was further affected by the separation from molecular systematists, who dismissed taxonomy as merely descriptive [19].

Turrill’s renaissance only began when the term “integrative taxonomy” was coined [20]. This concept defines an integrated approach to species delimitation that considers molecular, morphological, ecological, phylogenetic, and other comparative data together.

Integrative taxonomy brought molecular systematists back to taxonomy and the classification of living organisms. In addition, it allowed plant taxonomists to construct a more comprehensive understanding of biological diversity. More importantly, it provided a potential solution to the problems associated with applying only one of the many systematic species concepts available in the scientific literature.

Within the context of integrative taxonomy, a species is considered a hypothesis supported by multiple independent lines of evidence. Nowadays, the integrated approach is becoming common practice in plant systematics and has substantially contributed to solving problems in taxonomically critical groups characterized by long-standing taxonomic instability [21,22,23,24,25,26].

Despite this, the application of an integrated approach has introduced new topics of debate, mostly concerning species delimitation. The literature on this issue, which is strictly related to the problem of multiple species concepts, is complex, extensive, and well-documented [27,28].

In recent years, scholars have mostly focused on improving species delimitation methods based on molecular data [29,30,31], with other approaches allowing the integration of additional data types also being proposed [28,32,33,34]. In practice, however, molecular phylogenies often become the key, and sometimes the only, information used to delineate species, while other sources of evidence are overlooked.

Thus, the new challenge for scholars dealing with integrative taxonomy is to identify appropriate methods to incorporate non-molecular information into species delimitation, by standardizing how taxonomists integrate multiple independent lines of systematic evidence [35].

An attempt to integrate different sources of information into species concepts was proposed by Hong [28] through the so-called Gen-morph species concept. However, this approach applies only to sexually reproducing organisms and considers exclusively genetic and morphological evidence.

More recently, Oberprieler [36] developed a tool (the Wettstein tesseract) to guide taxonomic rank-attribution process at the species and subspecies level by standardizing the integration of systematic data. These data are represented by four categories: “genealogy”, “geography”, “ecology”, and “morphology”. All categories are expressed in binary form, with each considering the possible presence (=1) or absence (=0) of differences. For instance, cases of high morphological distinctiveness are interpreted as “1” for the “morphology” category, while cases of completely overlapping morphology are interpreted as “0”.

Despite being an easy and practical tool, the major limitation of the Wettstein tesseract lies in the impossibility of assigning a binary value to all cases. For example, cases of incompletely distinct morphologies, unresolved phylogenies, or partially overlapping geographical distributions, cannot be unambiguously categorized as either the absence or presence of differences. Moreover, the application of the Wettstein tesseract is also limited by its internal structure, which is constrained to only four categories. Although these four categories reflect the most common approaches used in plant integrative taxonomy, other important sources of information, such as cytosystematics, reproductive biology, and chemosystematics, are not considered.

Previously, Donegan [37] provided a tool with similar aims to Oberprieler [36], but based only on continuous values of differentiation between two allopatric populations. Such a tool can only be applied with quantitative data and when the geographical distributions of the tested populations do not overlap.

Thus, both tools [36,37] suffer from several limitations that restrict their wide diffusion and application by the scientific community. Consequently, a proper and general application of these tools to the millions of existing taxonomic hypotheses is impossible. Moreover, both approaches were developed primarily to support new taxonomic hypotheses rather than to extensively and retrospectively evaluate the solidity of a large number of species (i.e., already formulated taxonomic hypotheses).

To address these problems, we introduce here the idea of a standardized heuristic index that allows estimation of the degree of support for a taxonomic hypothesis based on the systematic data available in the literature. This index, named the Turrill’s Omega Index (TOI), aims to provide a transparent evaluation of the evidence supporting a given taxonomic hypothesis.

## 2. Description of the Turril’s Omega Index

Regardless of the species concept adopted and the complexity of the underlying evolutionary processes, systematists ultimately formulate taxonomic hypotheses based on putative biological discontinuities inferred from the available data. Each taxonomic hypothesis is supported (or, in the case of synonyms, not supported) to varying degrees by the systematic evidence available.

**Figure 1:**
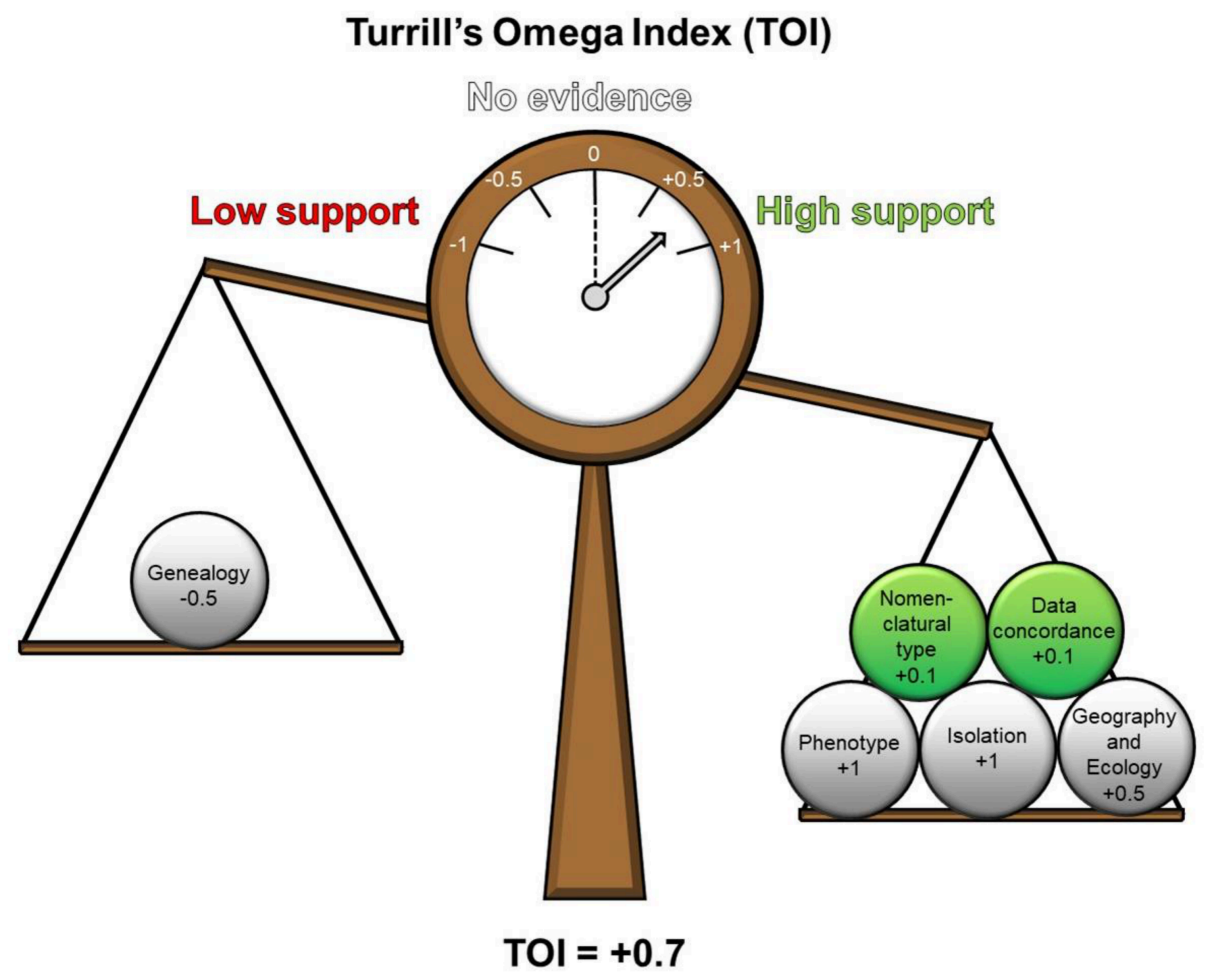
Schematic representation of the Turrill’s Omega Index as a sort of balance, able to summarize the overall degree of support of a taxonomic hypothesis. In this theoretical example, a typified species showing full morphological (Phenotype) and reproductive (Isolation) distinctiveness, allopatry (geography within Geography and Ecology), will result in an overall decisive support of a taxonomic hypothesis, despite the occurrence of a scarce genetic differentiation (Genealogy). Green indicates the possible adjustments associated with concordance between at least two of the three macro-categories (Phenotype, Genealogy, and Isolation) and with the presence of a designated nomenclatural type.

The Turrill’s Omega Index (TOI) is a numerical index designed to summarize the level of taxonomic support for a given taxon, based on the most common lines of evidence used in systematics. The underlying concept is effectively illustrated by the metaphor of a balance scale (Figure 1). Assuming a hypothetical condition of complete support (+1) and, conversely, complete lack of support (−1), a value of 0 represents a state of equilibrium. This indicates that the available evidence is insufficient to determine whether a given taxon is well supported or not. This neutral value may arise either from the absence of relevant information (i.e., no weights on either side of the balance) or from conflicting evidence, whereby some available data support the taxon as a hypothesis while other data do not. Intermediate values, such as +0.5 and −0.5, instead represent individual lines of evidence that provide only partial support or partial lack of support, respectively.

The basic idea underlying this index is to assign numerical scores to different lines of evidence, grouped in four key macro-categories of systematic data: Phenotype, Genealogy, Isolation, and Geography-Ecology. Each macro-category can include one or more sub-categories, depending also on the biological characteristics of the investigated organisms.

In the TOI exemplification presented here, we employed the most commonly used approaches for species delimitation in plant systematics [1,36]: morphology and chemosystematics within Phenotype; molecular systematics and cytosystematics within Genealogy; reproductive biology within Isolation; and geography and ecology within Geography-Ecology. Scoring criteria for each category used for this example of application are reported in Table 1.

To reduce the subjectivity among different scorers, we also provided more detailed guidelines for decision-making within each category (Table S1). For each category within Phenotype, Genealogy, and Isolation, as well as in the overall TOI index, scores vary between approximately −1 and +1. Overall, negative values indicate insufficient to absent support, whereas positive values indicate sufficient to strong support for a species hypothesis.

Values approaching zero never imply lack of support, but rather neutrality (e.g., no data available or contrasting evidence) and therefore the need for further studies. For each category within Geography-Ecology, where the absence of differences does not necessarily imply a lack of support (see, e.g., [38]), scores vary only between 0 and 1.

By first calculating the mean scores within each macro-category and then averaging the resulting macro-category scores, a single value (TOI) is obtained. In this calculation, each category is considered equally relevant and receives the same weight.

We acknowledge that, during the process of making taxonomic decisions, taxonomists do not necessarily assign equal importance to phylogenetic, morphological, or ecological data. However, our approach is not intended to *make* taxonomic decisions; rather, it provides a tool to summarize the available systematic evidence underlying an *already established and previously formulated* taxonomic decision. Moreover, molecular evidence should not always be interpreted as a direct proxy for species boundaries, since introgression, incomplete lineage sorting, hybridization, and polyploidization may generate incongruent genealogical patterns without undermining species validity [39,40]. Furthermore, several molecular species delimitation analyses have revealed population structure rather than species boundaries [41,42].

After averaging the score within and among macro-categories, slight adjustments are made to the final TOI value based on the concordance between macro-categories. Specifically, positive or negative concordance among at least two of the three macro-categories Phenotype, Genealogy, and Isolation results in adding or subtracting 0.1 from the final TOI value.

The rationale underlying this adjustment is as follows. To maintain the heuristic index simple and readily interpretable, evidence scores within each macro-category are first averaged, and the resulting mean scores of the four macro-categories are then averaged again to obtain the final TOI value. Although this approach provides a straightforward and transparent calculation, it may fail to adequately capture concordance among Phenotype, Genealogy, and/or Isolation macro-categories. Agreement among these categories, whether positive or negative, represents a particularly informative pattern [36] that should be appropriately weighted. Because the averaged scores of these macro-categories are typically low, applying a correction factor of +0.1 when evidence is concordantly positive, or −0.1 when evidence is concordantly negative, ensures that the index more accurately reflects the overall support for the taxonomic hypothesis under evaluation.

A simple hypothetical example illustrates this point. Consider a species showing partial morphological distinctiveness (+0.5), no chemosystematic information (0), partial phylogenetic distinctiveness (+0.5), no cytosystematic information (0), and no additional sources of evidence. The resulting scores would be +0.25 for Phenotype, +0.25 for Genealogy, 0 for Isolation, and 0 for Geography-Ecology. Without the concordance adjustment, the TOI would be +0.125, corresponding to *weak evidence for support: more systematic studies are needed* (see below). This occurs despite the fact that all available evidence consistently supports the taxonomic hypothesis. After applying the +0.1 correction, the TOI increases to +0.225, corresponding to *moderate evidence for support*. This value more appropriately reflects the level of support provided by the available data.

Consider now the same hypothetical species, but assume that lack of reproductive isolation with related species is demonstrated, corresponding to a score of −1 in the Isolation category. Without the concordance adjustment, the TOI would decrease to −0.125, corresponding to *moderate evidence for non-support*. However, this outcome does not reflect the consistent agreement between the morphological and phylogenetic evidence supporting taxonomic distinctiveness. By applying the correction, the TOI instead becomes −0.025, corresponding to *weak evidence for non-support: more systematic studies are needed*. This result more accurately represents a situation in which conflicting lines of evidence warrant caution rather than a conclusion against the taxonomic hypothesis.

Such adjustments do not in contrast with unbiased quantification; rather, they represents a way to better account for the concordance among different lines of evidence, which is intrinsically relevant in terms of overall systematic support [36]. Similarly, slight adjustments derive from the typification status of the name under evaluation. In the case of a typified name, a value of |0.1| is assigned and added (for positive TOI values) or subtracted (for negative TOI values) from the final TOI value (Figure 1). Most names currently in use, at least in Algae, Fungi, and Plants, were published before 1958 and lack a designated type. Therefore, they require typification according to the procedures established by the Code [43]. These thousands of currently untypified names are not fully reliable, as their application is not fixed. This represents an objective source of intrinsically higher or lower support for a given taxonomic hypothesis, and provides the rationale for this further adjustment.

After accounting for these adjustments and the possible values assigned to each category, TOI values can theoretically range from −0.95 to +1.2. To further explore the potential variation of the index, we simulated 100,000 TOI values by randomly assigning the scores and adjustments explained above.

The simulated TOI values range from −0.93 to +1.11, with distinct peaks suggesting a mixture of normal distributions. This is confirmed by the results of a model-based clustering approach using Gaussian mixture models [44,45], which identifies 10 different clusters. We used the mean values of these different curves as a baseline to identify different degrees of support for taxonomic hypotheses:

−0.95 < TOI < −0.46: decisive evidence for non-support
−0.46 < TOI < −0.32: very strong evidence for non-support
−0.32 < TOI < −0.23: strong evidence for non-support
−0.23 < TOI < −0.11: moderate evidence for non-support
−0.11 < TOI < +0.03: weak evidence for non-support: more systematic studies are needed
+0.03 < TOI < +0.14: weak evidence for support: more systematic studies are needed
+0.14 < TOI < +0.24: moderate evidence for support
+0.24 < TOI < +0.45: strong evidence for support
+0.45 < TOI < +0.63: very strong evidence for support
+0.63 < TOI < +1.20: decisive evidence for support

We present an example of TOI scoring and interpretation in 11 real cases concerning flowering plants endemic to the Italian flora, for which we have direct expertise and familiarity with the relevant literature (Table 2, see also Supplementary Material File S1 for more details [46,47,48,49,50,51,52,53,54,55,56,57,58,59,60,61,62,63,64,65,66,67,68,69,70,71,72,73,74]).

These species were selected because, based on our research experience and systematic knowledge, we could evaluate whether the obtained TOI values were able to properly summarize the available systematic information. Although these results cannot be considered a formal validation, the calculated TOI values consistently reflect the current taxonomic status of these taxa.

According to Bartolucci et al. [75] and subsequent updates, the strong to decisively supported *Aquilegia bertolonii* Schott and *A. lucensis* E.Nardi (Ranunculaceae), *Armeria arenaria* (Pers.) F.Dietr. subsp. *marginata* (Levier) Arrigoni (Plumbaginaceae), *Cardamine baldensis* Fritsch (Brassicaceae), *Crocus ilvensis* Peruzzi & Carta (Iridaceae)*, Leontodon montecristensis* Foggi, N.Kilian, Landi & Zoccola (Asteraceae), *Polygala flavescens* DC. subsp. *maremmana* (Fiori) Arrigoni (Polygalaceae), and *Siler montanum* Crantz subsp. *apuanum* F.Conti & Bartolucci (Apiaceae) are accepted taxa.

Conversely, the weakly non-supported *Hieracium elbanum* Belli ex Baroni (Asteraceae) and the weakly supported *Knautia gussonei* Szabó (Caprifoliaceae) are both preliminarily accepted but considered as “taxonomically doubtful” [75]. Finally, according to the same authors, the moderately non-supported *Rhaponticoides calabrica* Puntillo & Peruzzi (Asteraceae) is currently not accepted and is considered as a synonym of *R. centaurium* (L.) M.V.Agab. & Greuter.

## 3. Relevance and Future Impacts

The proposed index has the potential to make a strong and transformative impact in the fields of systematics and taxonomy. Based on systematic data available in the literature, or by incorporating of newly produced data, the degree of support for any taxonomic hypothesis can be easily summarized, regardless of the species concept adopted.

Accordingly, the long-standing debate surrounding the question “what is a species?”, which is unlikely to ever receive a universally accepted answer from a systematic perspective [7,76,77,78], may benefit from a more practical taxonomic approach provided by this index, which can serve as a useful complementary tool.

In addition, the availability of information concerning the degree of support for a species hypothesis could allow taxonomists and, especially, the users of taxonomy (e.g., ecologists, conservationists, biologists involved in applied research, and non-biologists) to critically consider these data rather than treating species names as unchanging factual units.

In case of positive TOI values, the users of taxonomy can be confident that the taxon under consideration is (more or less) supported. Conversely, negative values indicate that the taxon currently lacks support and may be synonymized in the future. Finally, values approaching zero indicate that additional systematic studies are needed to better clarify the taxonomic status of the species. The major strengths of our new index are that it is (1) all-inclusive, since it can be virtually applied to any taxonomic hypothesis and not only to specific cases; (2) easy to interpret, since it consists of a single continuous value; (3) standardized, allowing direct comparison among the levels of support of different species hypotheses.

Moreover, the value of TOI extends far beyond its general scientific relevance in systematic and taxonomic research, as it may play a pivotal role in supporting the prioritization of conservation efforts. Indeed, species names, besides representing taxonomic hypotheses, also serve as the basic unit for conservation practices.

As highlighted by Bortolus [79], the use of unreliable taxonomies can significantly hinder ecological studies and conservation actions. In the era of *triage* in nature protection [80], the conservation of poorly supported taxa may consume economic and human resources at the expense of threatened, well supported species [6].

Furthermore, the discrepancy between taxonomists’ and conservationists’/ecologists’ perspectives concerning species names may have negative implications for conservation [6]. To date, stakeholders and policymakers lack tools allowing them to obtain information on the degree of support for taxa.

As a consequence, among taxa that are equally threatened according to standard conservation criteria (e.g., the IUCN Red List assessment protocol or the Endangered Species Act in the USA), conservation funds may be allocated to weakly supported species that subsequent studies could relegate to synonymy. These resources could instead support equally threatened, better-supported species that may warrant higher conservation priority. In this context, TOI provides an additional source of information to support more informed conservation decisions, complementing rather than replacing existing conservation assessment frameworks.

A further impact concerns how taxonomy users perceive taxonomic changes. Indeed, such changes are often considered unnecessary and destabilizing [2,3,81]. By implementing of TOI into databases, researchers or amateurs working with scientific names (e.g., compiling taxonomic checklists of a given territory or studying ecological communities) could better understand the amount and type of data supporting a taxonomic change.

To guarantee the general usability of the new index, public availability of the obtained data is crucial for disseminating and replicating calculated TOIs, including the scores to each data category.

The relevance of having a single proxy value summarizing the support for a taxonomic hypothesis (i.e., the TOI value) is particularly important for taxonomy users, who may not be able or willing to examine in detail the support (or lack of support) provided by each category of data.

Conversely, for professional taxonomists, the individual scores assigned to each category may be even more useful and informative, particularly for planning further research where needed. For this reason, we envisage the future integration of datasets containing all available information into public online nomenclatural and taxonomic portals, rather than limiting these resources to TOI values alone.

This goal could be achieved through the development on online systematic databases designed to store and make available all retrieved data. Such databases could be easily integrated with, or made interoperable with, major nomenclatural and taxonomic databases (for land plants, see for instance Borsch et al. [82]).

These repositories could readily integrate this information, provided that the scientific community recognizes its value, as we hope, and that collective global collaborative efforts are undertaken in this regard.

Among the general concepts recently highlighted as necessary for governing and assessing the quality of global species lists, authors mentioned criteria such as “taxon recognition” [83] and “treatment authority” [84]. These criteria are closely related to what is quantified by TOI, which could substantially enrich global species lists and improve the overall understanding of these issues.

In this envisioned scenario, users searching for a name would not only find the currently accepted name, but also the corresponding TOI value. This value would summarizes the degree of support that the name holds as a scientific hypothesis and explain how this numerical evaluation was derived.

## 4. Limitations and Future Developments

By following the guidelines developed for score attribution (see Table S1), we expect that different taxonomists should derive comparable TOI values when evaluating the same taxon. However, because a certain degree of subjectivity is inevitably associated with expert judgement, future calibration exercises involving multiple independent experts could help assess the consistency, reproducibility, and repeatability of the proposed index. Moreover, the current theoretical validation is encouraging but remains limited. Future applications across different organismal groups and a broader range of evolutionary scenarios will strengthen confidence in the proposed thresholds.

## Figures and Tables

**Table 1:** Scoring criteria for each systematic category, organized in macro-categories, employed to calculate TOIs in the example presented concerning selected vascular plant species. Either positive or negative concordance among at least two of the three macro-categories Phenotype, Genealogy, and Isolation implied adding/removing 0.1 from the final TOI value. Also typification, if present, was scored as |0.1| and added (in case of positive TOI values) or removed (in case of negative TOI values) from the final TOI value. See the guidelines in Table S1 for more details.

Data Type	Scoring Criteria
**Phenotype**	
Morphology	−1: Full morphological overlap with related taxa −0.5: Slight morphological differences resulting in a strong overlap 0: Contrasting results/no information available/flawed or unclear methodology +0.5: Presence of some significant morphological differences but still partial overlap +1: No morphological overlap
Chemosystematics	−1: Chemical profile overlaps completely with related taxa −0.5: Minor, not statistically significant, differences with partial overlap 0: Contrasting results/no information available/flawed or unclear methodology +0.5: At least some significant difference in chemical markers +1: More data suggesting full and consistent differences in chemical markers
**Genealogy**	
Molecular Systematics	−1: Analyses point to a polyphyletic group or to a group not discernible from related taxa (also considering DNA barcodes) −0.5: The group is scarcely differentiated from related taxa 0: Contrasting results/no information available/flawed or unclear methodology +0.5: The group shows moderate support or there is evidence of slight admixture (also considering DNA barcodes) +1: Analyses point to a completely diverging, well supported, group and/or with distinct DNA barcodes
Cytosystematics	−1: No significant difference in cytogenetic features and full overlap of values −0.5: No significant difference in cytogenetic features and partial overlap of values 0: Contrasting results/no information available/flawed or unclear methodology +0.5: A least a single significant difference in cytogenetic features +1: Significant differences in cytogenetic features with no overlap
**Isolation**	
Reproductive Biology	−1: Full interfertility with related species −0.5: Preliminary reproductive studies show no significant isolation 0: Contrasting results/no information available/flawed or unclear methodology +0.5: At least some significant reproductive isolation +1: More data suggesting full and consistent reproductive isolation
**Geography and Ecology**	
Geography	0: Sympatry with related taxa or no data on the species distribution +0.5: Parapatry with related taxa +1: Allopatry with related taxa
Ecology	0: Ecological requirements overlap completely with related species or no ecological characterization available +0.5: Partial overlap of statistically significant niche differences and/or qualitative assessment of the specie ecology +1: No overlap of statistically significant niche differences

**Table 2:** Examples of TOI values calculated in 11 selected flowering plant species/subspecies. Phenotype: MOR (Morphology), CHE (Chemosystematics); Genealogy: MOL (Molecular systematics), CYT (Cytosystematics); Isolation: REP (Reproductive biology); Geography and Ecology: GEO (Geography), ECO (Ecology). type: Nomenclatural type, con: concordance of data among at least two of the three macro-categories Phenotype, Genealogy, and Isolation. TOI values are highlighted in **bold**. Well-supported taxa have a green background, weakly supported taxa (TOI values close to 0) a yellow background, and unsupported taxa a red background.

Taxon	MOR	CHE	MOL	CYT	REP	GEO	ECO	Mean	con	Type	TOI	Interpretation
*Aquilegia bertolonii* Schott	+0.5	0	+1	0	0	+1	+0.5	+0.375	+0.1	+0.1:	**+0.575**	very strong support
*Aquilegia lucensis* E.Nardi	+0.25	0	0	0	0	+1	+0.5	+0.219	0	+0.1	**+0.319**	strong support
*Armeria arenaria* (Pers.) F.Dietr. subsp. *marginata* (Levier) Arrigoni	+1	0	+0.5	0	0	+1	+0.5	+0.375	+0.1	+0.1	**+0.575**	very strong supported
*Cardamine baldensis* Fritsch	+1	0	+0.5	0	0	+1	0	+0.313	+0.1	+0.1	**+0.513**	very strong support
*Crocus ilvensis* Peruzzi & Carta	+1	+1	+1	+0.5	0	+1	0	+0.563	+0.1	+0.1	**+0.763**	decisive support
*Hieracium elbanum* Belli ex Baroni	0	0	0	0	0	+1	0	+0.125	0	−0.1	**−0.025**	weak non-support
*Knautia gussonei* Szabó	0	0	−1	0	0	+1	0	0	0	+0.1	**+0.100**	weak support
*Leontodon montecristensis* Foggi, N.Kilian, Landi & Zoccola	+0.5	0	+1	+0.5	0	+1	0	+0.375	+0.1	+0.1	**+0.575**	very strong support
*Polygala flavescens* DC. subsp. *maremmana* (Fiori) Arrigoni	+1	+1	0	0	0	+1	0	+0.375	0	+0.1	**+0.475**	very strong support
*Rhaponticoides calabrica* Puntillo & Peruzzi	−0.25	0	0	0	0	0	0	−0.031	0	−0.1	**−0.131**	moderate non-support
*Siler montanum* Crantz subsp. *apuanum* F.Conti & Bartolucci	+1	0	0	0	0	+1	0	+0.250	0	+0.1	**+0.350**	strong support

## Data Availability

All data generated or analyzed during this study are included in this published article and its Supplementary Information Files.

## Supplementary Materials

The following supporting information can be downloaded at https://www.mdpi.com/article/10.3390/biology15151265/s1, Table S1: Scoring guidelines for each systematic category, organized in macro-categories, employed to calculate TOIs in the example presented concerning selected vascular plant species. Either positive or negative concordance among at least two of the three macro-categories Phenotype, Genealogy, and Isolation implied adding/subtracting 0.1 from the final TOI value. Also typification, if present, was scored as |0.1| and added (in case of positive TOI values) or subtracted (in case of negative TOI values) from the final TOI value; Supplementary Material File S1. Example of a dataset including TOI scoring and interpretation in 11 real cases concerning flowering plants endemic to the Italian flora.
