# Supplementary material for: Turrill’s Omega: A Heuristic Index for Quantifying the Support of a Taxonomic Hypothesis
Source: Biology (Basel). 2026 Aug 2;15(15):1265. doi: 10.3390/biology15151265 (PMC13464593; doi:10.3390/biology15151265)
Supplement: Supplementary file 1 [file biology-15-01265-s001.zip › biology-4460498-supplementary-Table S1.pdf]

Table S1. Scoring guidelines for each systematic category, organized in macro-categories, employed to calculate TOIs in the example presented concerning selected vascular plant species. Either positive or negative concordance among at least two of the three macro-categories Phenotype, Genealogy, and Isolation implied adding/subtracting 0.1 from the final TOI value. Also typification, if present, was scored as |0.1| and added (in case of positive TOI values) or subtracted (in case of negative TOI values) from the final TOI value.

Data type	Scoring guidelines
**Phenotype**	
Morphology	**–1: Full morphological overlap with related taxa**<br>Study presenting a multivariate analysis (e.g., PCA, PCoA, LDA, FAMD, clustering) in which the evaluated taxon overlaps completely. To be checked: confidence ellipses if present, confusion matrices, BIC in model-based clustering with other related taxa.<br>Study presenting overlapping boxplots/means and standard deviation ranges, non-significant differences among the evaluated taxon and related taxa for one or more diagnostic characters.<br><br>**–0.5: Slight morphological differences resulting in a strong overlap**<br>Study presenting a multivariate analysis (e.g., PCA, PCoA, LDA, FAMD, clustering) in which the evaluated taxon somewhat overlaps. To be checked: confidence ellipses if present, confusion matrices, BIC in model-based clustering with other related taxa. No clear distinction.<br>Study presenting not completely overlapping boxplots/means and standard deviation ranges, non-significant differences among the evaluated taxon and related taxa for one or more diagnostic characters.<br><br>**0: Contrasting results / no information available / flawed or unclear methodology**<br>This score can be attributed to a taxon for which: different studies presenting comparative morphological data are contrasting; no information is available; the method used to produce the comparative data is flawed or unclear.<br>Study presenting only qualitative morphological data, with no quantitative statistical analyses, including the case in which only a qualitative morphological description is available only in the protologue.<br><br>**+0.5: Presence of some significant morphological differences but still partial overlap**<br>Study presenting a multivariate analysis (e.g., PCA, PCoA, LDA, FAMD, clustering) in which the evaluated taxon somewhat overlaps. To be checked: confidence ellipses if present, confusion matrices, BIC in model-based clustering with other related taxa. No clear distinction.<br>Study presenting not completely overlapping boxplots/means and standard deviation/ranges and significant differences among the evaluated taxon and related taxa for one or more diagnostic characters. If effect size are available (e.g. Cohen's d) a value < 1.<br><br>**+1: No morphological overlap**<br>Study presenting a multivariate analysis (e.g., PCA, PCoA, LDA, FAMD, clustering) in which the evaluated taxon completely separated. To be checked: confidence ellipses if present, confusion matrices, BIC in model-based clustering with other related taxa.<br>Study presenting non overlapping boxplots/means and standard deviation/ranges and significant differences among the evaluated taxon and related taxa for one or more diagnostic characters. If effect size is available (e.g. Cohen's d) a value > 1.

Chemosystematics	**−1: Chemical profile overlaps completely with related taxa**<br>No compounds allowing the phytochemical discrimination of the evaluated taxon in respect with related taxa. The phytochemical profile (inferred from multivariate analyses) of the evaluated taxon completely overlaps with the ones of related taxa.<br><br>**−0.5: Minor, not statistically significant, differences with partial overlap**<br>No compounds allowing the phytochemical discrimination of the evaluated taxon in respect with related taxa. The phytochemical profile (inferred from multivariate analyses) of the evaluated taxon completely is partially overlapping from the ones of related taxa.<br><br>**0: Contrasting results / no information available / flawed or unclear methodology**<br>This score can be attributed to a taxon for which: different studies presenting data on comparative phytochemistry are contrasting; no information is available; the method used to produce these comparative data is flawed or unclear.<br><br>**+0.5: At least some significant difference in chemical markers**<br>At least one unique compound allowing the phytochemical discrimination of the evaluated taxon in respect with related taxa. The phytochemical profile (inferred from multivariate analyses) of the evaluated taxon partially overlaps with the ones of related taxa.<br><br>**+1: More data suggesting full and consistent differences in chemical markers**<br>Some unique compounds allowing the phytochemical discrimination of the evaluated taxon in respect with related taxa. The phytochemical profile (inferred from multivariate analyses) of the evaluated taxon is completely distinct from the ones of related taxa.
**Genealogy**	
Molecular Systematics	**−1: Analyses point to a polyphyletic group or to a group not discernible from related taxa**<br>Phylogenetic and/or phylogenomic trees and/or networks point to the evaluated taxon being polyphyletic and/or being nested within other clades representing related taxa. If the accessions of the evaluated taxon belong to a single clade, the bootstrap and/or posterior probability values are very low (approximately below 50 and/or 0.5, respectively).<br>Population genetics and/or genomics molecular data point to the evaluated taxon having no genetic differentiation with related taxa: overlap in an ordination analysis (e.g., PCA, t-SNE, UMAP), individuals the same taxon belonging to different genetic groups retrieved from clustering approaches (e.g., STRUCTURE analyses, ADMIXTURE analyses, DAPC).<br>If the support of different grouping scenarios is tested with multi-species coalescent model (MSC) based approaches (e.g., BPP, BFD) the current taxonomy has a support (expressed in term BIC, BF, MLE etc.) lower than alternative grouping hypotheses.<br>A group not discernible from related taxa based on DNA barcodes.<br><br>**−0.5: The group is scarcely differentiated from related taxa**<br>Phylogenetic and/or phylogenomic trees and/or networks point to the evaluated taxon being nested within other clades representing related taxa. The node underlying the accessions of evaluated taxon's clade has a low bootstrap and/or posterior probability value (ranging approximately from 50 to 70 and/or from 0.5 to 0.7, respectively).

Population genetics and/or genomics molecular data point to the evaluated taxon having low (≤ 2%) genetic differentiation with related taxa: slight separation in an ordination analysis (e.g., PCA, t-SNE, UMAP), individuals the same taxon belonging to different genetic groups retrieved from clustering approaches (e.g., STRUCTURE analyses, ADMIXTURE analyses, DAPC).
If the support of different grouping scenarios is tested with multi-species coalescent model (MSC) based approaches (e.g., BPP, BFD) (expressed in term BIC, BF, MLE etc.) the has a support lower than alternative grouping hypotheses.
A group widely overlapping with related taxa based on DNA barcodes (e.g., partial, but not effective distinction).

0: Contrasting results / no information available / flawed or unclear methodology
This score can be attributed to a taxon for which: different approaches/markers used in molecular systematics are contrasting; no information is available; the method used to produce these comparative data is flawed or unclear.

+0.5: The group shows moderate differentiation
Phylogenetic and/or phylogenomic trees and/or networks point to the accessions of the evaluated taxon belonging to a different clade in comparison to related taxa; the node underlying that clade has a moderate bootstrap and/or posterior probability value (ranging approximately from 70 to 95 and/or from 0.7 to 0.95, respectively).
Population genetics and/or genomics molecular data point to the evaluated taxon having moderate (> 2%) genetic differentiation with related taxa: separation in an ordination analysis (e.g., PCA, t-SNE, UMAP), individuals the same species belonging to a single genetic groups retrieved from clustering approaches (e.g., STRUCTURE analyses, ADMIXTURE analyses, DAPC) with no or slight admixture with other groups.
If the support of different grouping scenarios is tested with multi-species coalescent model (MSC) based approaches (e.g., BPP, BFD) the support (expressed in term BIC, BF, MLE etc.) of current taxonomy shows one of the highest values.
A group only partially discernible from related taxa based on DNA barcodes (e.g., some markers are effective, but not consistently across all populations).

+1: Analyses point to a completely diverging, well supported, group
Phylogenetic and/or phylogenomic trees and/or networks point to the accessions of the evaluated taxon belonging to a different, well supported clade in comparison to related taxa; the node underlying that clade has a high bootstrap and/or posterior probability value (higher than 95 and/or than 0.95, respectively).
Population genetics and/or genomics molecular data point to the evaluated taxon having high genetic differentiation (> 15%) with related taxa: clear separation an ordination analysis (e.g., PCA, t-SNE, UMAP), individuals the same species belonging to a single genetic groups retrieved from clustering approaches (e.g. STRUCTURE analyses, ADMIXTURE analyses, DAPC), although with slight admixture with other groups.
If the support of different grouping scenarios is tested with multi-species coalescent model (MSC) based approaches (e.g., BPP, BFD) (expressed in term BIC, BF, ML etc.) the current taxonomy is be the best supported scenario.
A group fully discernible from related taxa based on DNA barcodes (i.e. one or more markers fully effective).

Cytosystematics	**−1: No significant difference in cytogenetic features**<br>No differences in chromosome number, no significant differences in karyotype symmetry (e.g., M_{CA}, CV_{CL}) among the evaluated taxon and related taxa. No significant differences in genome size among the evaluated taxon and related taxa. Same banding patterns among the evaluated taxon and related taxa.<br><br>**−0.5: No significant difference in cytogenetic features and partial overlap of values**<br>No differences in chromosome number among the evaluated taxon and related taxa. Karyotype symmetry (e.g., M_{CA}, CV_{CL}) is partially overlapping and not significantly different among the evaluated taxon and related taxa. No significant differences in genome size, with partially overlapping values among the evaluated taxon and related taxa. Very similar banding patterns among the evaluated taxon and related taxa.<br><br>**0: Contrasting results / no information available / flawed or unclear methodology**<br>This score can be attributed to a taxon for which: different studies presenting data on comparative cytogenetics are contrasting; the chromosome number is the sole information available and is identical to those of related taxa; no information is available; the method used to produce these comparative data is flawed or unclear.<br><br>**+0.5: A least a single significant difference in cytogenetic features**<br>No differences in chromosome number, but significant differences among the evaluated taxon and related taxa in at least one of the following cytogenetic pieces of evidence. Significant difference in karyotype symmetry indexes (e.g., M_{CA}, CV_{CL}). Significant difference in genome size among the evaluated taxon and related taxa. Different banding patterns among the evaluated taxon and related taxa.<br><br>**+1: Significant differences in cytogenetic features with no overlap**<br>Stable differences in chromosome number (i.e. polyploidy or dysploidy) within the evaluated taxon and different from related taxa. Significant differences in karyotype symmetry indexes (e.g., M_{CA}, CV_{CL}) with no overlap among the evaluated taxon and related taxa. Significant differences in genome size, with no overlap of values among the evaluated taxon and related taxa. Very different banding patterns among the evaluated taxon and related taxa.
**Isolation**	
Reproductive Biology	**−1: Full interfertility with related species**<br>This score can be attributed if multiple experiments and observations have been carried out on reproductive biology of the evaluated taxon. Flowering period of the evaluated taxon completely overlaps with related taxa. No mechanical constraints to heterologous pollination between the evaluated taxon and related taxa: no differences in flower morphology, pollinators. Pollinator spectrum of the evaluated taxon is shared by related taxa. Pollen of the evaluated taxon germinate on the stigma of related taxa, and the pollen tube can reach and fertilize the ovules.<br>Crossing experiments show a full interfertility among the evaluated taxon and related taxa, with fertile offsprings, without declining fitness (either in terms of zygote or embryo development, seed germination, etc.), across generations.<br><br>**−0.5: Preliminary reproductive studies show no significant isolation**<br>This score can be attributed if only few or preliminary experiments/observations have been carried out on reproductive biology of the evaluated taxon, obtaining results similar to those of the previous score.

	**0: Contrasting results / no information available / flawed or unclear methodology**<br>This score can be attributed to a taxon for which: different approaches presented in the study on reproductive biology are contrasting; no information is available; the method used to produce these data is flawed or unclear.<br><br>**+0.5: At least some significant reproductive isolation**<br>This score can be attributed if only few or preliminary experiments/observations have been carried out on reproductive biology of the evaluated taxon, obtaining results similar to those of the following score. Multiple studies show only partial interfertility.<br><br>**+1: More data suggesting full and consistent reproductive isolation**<br>This score can be attributed if multiple experiments and observations have been carried out on reproductive biology of the evaluated taxon. The flowering period of the evaluated taxon does not overlap with related taxa. There are mechanical constraints to pollination between the evaluated taxon and related taxa: differences in flower morphology, pollinators. Pollinator spectrum of the evaluated taxon is not, or only partially, shared by related taxa. Pollen of the evaluated taxon do not germinate easily on the stigma of related taxa, and the pollen tube cannot reach the ovules.<br>Crossing experiments show no fertility among the evaluated taxon and related taxa, with sterile offsprings, declining fitness (in terms of zygote or embryo developments, seed germination) across generations.
**Geography and Ecology**	
Geography	**0: Sympatry with related taxa or no data on the species distribution**<br>Distribution data obtained from herbarium specimens, online databases (e.g., GBIF, iNaturalist, etc.), literature (e.g., regional or local floras, protologue) point to a complete overlap of the distribution range of evaluated taxon with the ones of related taxa. This score can be assigned also if there are no data on the distribution of the evaluated taxon. However, this score also can be assigned if its distribution on a national/regional level (from POWO, Euro+Med Plantbase, etc.) is known and overlaps with that of related taxa.<br><br>**+0.5: Parapatry with related taxa**<br>Distribution data obtained from herbarium specimens, online databases (e.g., GBIF, iNaturalist, etc.), literature (e.g., regional or local floras, protologue) shows that the distribution edges of the evaluated taxon overlap with those of the ranges of related taxa, showing one or many contact zones / intermingled areas. However, this score can be assigned also if there are no data on the distribution of the evaluated taxon, but its distribution on a national/regional level (from POWO, Euro+Med Plantbase, etc.) is known and the countries in which the evaluated taxon occurs are contiguous with those in which related taxa occur.<br><br>**+1: Allopatry with related taxa**<br>Distribution data obtained from herbarium specimens, online databases (e.g., GBIF, iNaturalist, etc.), literature (e.g., regional or local floras, protologue) shows that the ranges of the evaluated taxon and those of related taxa are not overlapping; this score can also be attributed if evident geographical barriers can be identified. Finally, this score can be assigned also if there are no data on the distribution of the evaluated taxon, but its distribution on a national/regional level (from POWO, Euro+Med Plantbase, etc.) is known and the countries in which the evaluated taxon occurs do not border those in which related taxa occur.

Ecology	**0: Ecological requirements overlap completely with related species or no ecological characterization available**<br>This score can be attributed to taxa whose modelled ecological niches overlap with related taxa (e.g., Schoener's D ranging from 0.5 to 1) and niche similarity test is non-significant. No significant differentiation in functional traits: same CSR strategy, no differences in single functional traits (e.g., SLA, plant height, stomatal conductance, photosynthetic efficiency, etc.), no differences in germination ecology (same germination response, same dormancy strategy, etc.). No differences in qualitative ecological assessments: same geological substrate, same habitats, same elevation ranges.<br><br>**+0.5: Partial overlap of statistically significant niche differences and/or qualitative assessment of the specie ecology**<br>This score can be attributed to taxa whose modelled ecological niches moderately overlap with related taxa (e.g., Schoener's D ranging from 0.2 to 0.5) and niche similarity test is either significant or not significant (if even lower). Slight differentiation in functional traits: CSR strategy, ranges only partially overlapping and means/median significantly different in one or more functional traits (e.g., SLA, plant height, stomatal conductance, photosynthetic efficiency, etc.), partial overlap in germination ecology (germination response, dormancy strategy, etc.). Finally, this score can also be attributed to a taxon for which only differences in qualitative ecological assessments are known: different geological substrate, different habitats, different elevation ranges.<br><br>**+1: No overlap of statistically significant niche differences**<br>This score can be attributed to taxa whose modelled ecological niches show low or null overlap with related taxa (e.g. Schoener's D ranging from 0 to 0.2) and niche similarity test significant. Clear differentiation in functional traits: CSR strategy, ranges not overlapping and means/median significantly different in one or more functional traits (e.g., SLA, plant height, stomatal conductance, photosynthetic efficiency etc.), no overlap in germination ecology (different germination response, different dormancy strategy, etc.). Quantitative differences in ecological assessments: different geological substrate, different habitats, different elevation ranges.
